# Sicily statement on classification and development of evidence-based practice learning assessment tools

**DOI:** 10.1186/1472-6920-11-78

**Published:** 2011-10-05

**Authors:** Julie K Tilson, Sandra L Kaplan, Janet L Harris, Andy Hutchinson, Dragan Ilic, Richard Niederman, Jarmila Potomkova, Sandra E Zwolsman

**Affiliations:** 1 Division of Biokinesiology and Physical Therapy, University of Southern California, Los Angeles, CA, USA; 2 Doctoral Programs in Physical Therapy, Dept. of Rehabilitation and Movement Sciences, University of Medicine and Dentistry of New Jersey, Newark, NJ, USA; 3 School of Health & Related Research, University of Sheffield, UK; 4 National Prescribing Centre, National Institute for Health and Clinical Excellence, Liverpool, UK; 5 Department of Epidemiology & Preventive Medicine, School of Public Health & Preventive Medicine, Monash University, VIC, Australia; 6 Center for Evidence-Based Dentistry, The Forsyth Institute, Boston, MA, USA; 7 Palacky University Medical Library, Olomouc, Czech Republic; 8 Department of General Practice/Family Medicine, Academic Medical Center-University of Amsterdam, the Netherlands

## Abstract

**Background:**

Teaching the steps of evidence-based practice (EBP) has become standard curriculum for health professions at both student and professional levels. Determining the best methods for evaluating EBP learning is hampered by a dearth of valid and practical assessment tools and by the absence of guidelines for classifying the purpose of those that exist. Conceived and developed by delegates of the Fifth International Conference of Evidence-Based Health Care Teachers and Developers, the aim of this statement is to provide guidance for purposeful classification and development of tools to assess EBP learning.

**Discussion:**

This paper identifies key principles for designing EBP learning assessment tools, recommends a common taxonomy for new and existing tools, and presents the Classification Rubric for EBP Assessment Tools in Education (CREATE) framework for classifying such tools. Recommendations are provided for developers of EBP learning assessments and priorities are suggested for the types of assessments that are needed. Examples place existing EBP assessments into the CREATE framework to demonstrate how a common taxonomy might facilitate purposeful development and use of EBP learning assessment tools.

**Summary:**

*The widespread adoption of EBP into professional education requires valid and reliable measures of learning. Limited tools exist with established psychometrics. This international consensus statement strives to provide direction for developers of new EBP learning assessment tools and a framework for classifying the purposes of such tools*.

## Background

"No single assessment method can provide all the data required for judgment of anything so complex as the delivery of professional services by a successful physician." [1]

Evidence-based practice (EBP), the integration and implementation of best available evidence with clinical expertise and patients' values and circumstances,[2] is a foundation for healthcare education across disciplines and international borders and has become an essential requirement for certification and re-certification in many health professions. Assessing EBP learning is hampered, however, by a relative dearth of validated and practical assessment tools [3]. Although the most recent systematic review[4] identified 104 unique EBP assessment tools, the majority of these tools have not been validated and address only limited constructs of EBP [4-6].

The aim of this consensus statement is to provide guidance for purposeful classification and development of EBP assessment tools. It highlights principles to be considered during tool development, provides a framework for classifying EBP assessment tools, and identifies the types of tools that are needed to promote more consistent evaluation of EBP teaching outcomes. This consensus statement also proposes principles and priorities for future efforts in tool development. It does not evaluate the effectiveness of different educational approaches in promoting evidence-based behaviour change and quality improvement.

## Methods

This statement was conceived by the delegates of the 5^th ^International Conference of Evidence-Based Health Care (EBHC) Teachers and Developers held in Sicily in October 2009 (http://www.ebhc.org) at its final plenary session. The initial structure of the statement and manuscript was developed following two conference round table discussions (4 hours) on how to classify and develop EBP learning assessment tools. Each author selected a section of the manuscript to research and develop, and their submissions were organized to focus the paper. An iterative process of shaping occurred through 4 conference calls and 7 draft reviews of the manuscript. The authors solicited input on the statement from non-author EBHC conference delegates in 2 phases - first from the conference steering committee (n = 12) and second from conference delegates (n = 66). Seventeen non-author conference attendees (22%) responded to the survey. Responses were incorporated into the final statement which was approved by author consensus. Authors and survey respondents constitute 25 individuals from 12 countries and 7 healthcare professions.

## Discussion

### Part I: Principles to consider when developing assessment tools

#### Categories of educational assessment

Educators can assess different dimensions of EBP learning, including reaction to initial exposure to EBP, knowledge attainment, or the ability to use EBP skills to improve patient care. The challenge of assessing healthcare professionals' learning is not unique to EBP. Numerous educators have contributed to defining the complex nature of assessing professional competencies like EBP. Table 1 illustrates our proposal for categorizing assessment of EBP educational outcomes.

**Table 1:** Categories of EBP Learner Educational Assessments

	Assessment Category	Example of what is assessed
1	Reaction to the EBP educational experience	Did the learners feel that the EBP educational experience provided benefit?

2	Attitudes about EBP	Do the learners value EBP as an important part of their role in healthcare?

3	Self-efficacy for conducting EBP	Do the learners have confidence in their ability to carry out the 5-step EBP process?

4	Knowledge about EBP principles	Do the learners know which study design is most appropriate for a prognostic study of a common condition?

5	Skills for performing EBP	Are the learners effective at conducting a PubMed search for systematic reviews?

6	Behaviour congruent with EBP as part of patient care	Do the learners identify knowledge gaps and pursue best available evidence to address them?

7	Benefit to Patients associated with EBP	Do the learners' EBP actions result in improved patient outcomes?

Modified from Freeth et al.'s Model of Outcomes of Interprofessional Education[7]

The proposed model is based upon work by Freeth et al. [7] Freeth and colleagues used their systematic review of interprofessional learning in healthcare to develop categories specific to assessment of healthcare education based upon those originally proposed in Kirkpatrick's Hierarchy of Levels of Evaluation [8]. We believe that these categories are well suited for assessing EBP learning because they allow educators to classify the impact of an EBP educational intervention from the most proximal phenomenon (the learners' experiences) to the most distal (patient care outcomes).

#### Linking assessment to learning aims and learner audiences

Tools for assessing the effectiveness of teaching EBP need to reflect the aims of the curriculum. Learning aims will ideally be matched to the needs and characteristics of the learner audience. Straus et al. [9] classified three unique EBP learner aims: to 'replicate' the EBP of others, to 'use' evidence summaries for EBP, and to 'do' the more time-intensive 5 steps of EBP defined by Dawes et al. in the original Sicily Statement [2]. Students, for example, may need a comprehensive grounding in the 5 steps of EBP, whereas health professionals who manage services may require skills in using evidence summaries. Educators need to match assessment tools to the unique learning aims of different learner groups. For example, a skills assessment of EBP 'users' may need to focus on ability to find and appropriately apply rigorously pre-appraised evidence. Conversely, a skills assessment of EBP 'doers' may need to focus on ability to find, critically appraise, and apply primary research [9].

The context and motivations for teaching, learning, and using EBP also need to be considered during assessment. Ability to master EBP skills can be influenced by contextual elements such as personal beliefs, organization barriers, variations in learning styles, and prior exposure to EBP. Motivations for using EBP also influence assessment. Practitioners learning to apply EBP in clinical practice may need to be assessed differently than undergraduate students, administrators, payers, and policy makers [10,11]. Likewise, assessment of EBP learning in the context of interprofessional education models may require special consideration [12]. Thus, developers of EBP assessment tools are encouraged to explicitly identify the type of learner and learning aims that a tool is designed to assess.

#### Objective of the assessment

Broadly speaking, the objective of an assessment can be one of two types - formative or summative. Formative assessment provides learners and teachers with information about competency development concurrent with the learning process, and can be used to influence the educational process and facilitate competence in real time. Summative assessments are used to establish competence or qualification for advancement. The potential high-stakes nature of summative assessments demand a greater degree of psychometric rigor compared to formative assessments [13]. EBP assessment developers should clearly articulate the objectives of EBP assessment tools to signpost their utility for different learners and learning aims.

### Part II: Framework for describing EBP learning assessment tools

**Figure 1:**
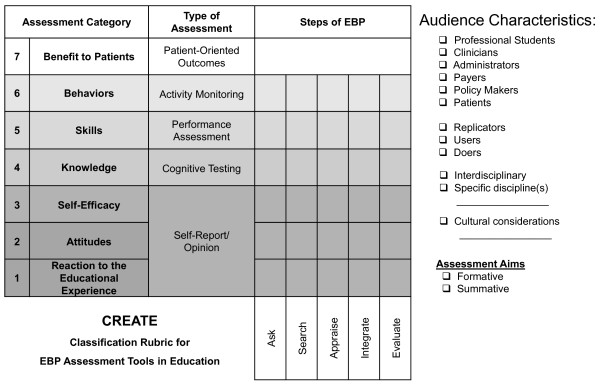
**The CREATE Framework**. The Classification Rubric for EBP Assessment Tools in Education (CREATE) is a framework for classifying EBP learner assessment tools. Assessment tool developers can use the CREATE framework by 'checking' the boxes in the grid that represent the assessment category (or categories) and step (or steps) of EBP assessed by their tool. Additionally the audience characteristics and assessment aims for which the assessment tool is intended can be indicated in the right hand column.

**Figure 2:**
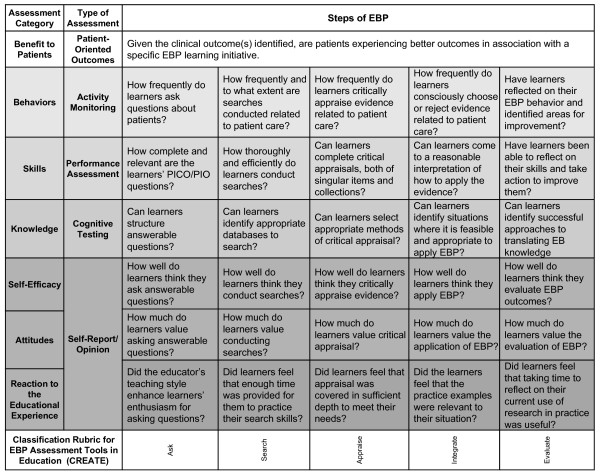
**Examples of the types of questions addressed in the CREATE framework**.

We propose the Classification Rubric for EBP Assessment Tools in Education (CREATE; Figure 1) for classifying EBP learner assessment tools. Using this framework, the nature of an assessment can be characterized with regard to the 5-step EBP model, type(s) and level of educational assessment specific to EBP, audience characteristics, and learning and assessment aims. In contrast to Table 1, the assessment categories are listed in reverse order to illustrate how one category may build on the next from most simple (Reaction to the Educational Experience) to most complex (Benefit to Patients). The type of assessment generally used for each category is also described. Sample questions for each element of the CREATE framework are provided in Figure 2. To facilitate consistent use of the CREATE tool, descriptions, examples when available, and discussion of each level of assessment follow.

#### Assessment Category

##### Reaction to the Educational Experience

In the CREATE framework, *Reaction to the Educational Experience *refers to learners' perspectives about the learning experience, including structural aspects (e.g. organization, presentation, content, teaching methods, materials, quality of instruction) and less tangible aspects such as support for learning [7]. These aspects represent potential covariates for the efficacy of an educational intervention [14], providing important information for educators and course designers although they are not direct measures of learning. Assessment of learner's reaction to an educational intervention is common in practice. For example, the Student Instructional Report II is a generic university-level teaching assessment developed by the Educational Testing Service that assesses learner reaction to education experiences [15]. We were not able to identify formally validated tools for this level of assessment specific to EBP learning. Learner reaction to an educational experience can be assessed through surveys that use questions appropriate to teaching different EBP steps, such as:

• *Did the instructor's teaching style enhance your enthusiasm for asking questions during ward rounds? (Ask)*

• *Was the lecture on literature searching at an appropriate level for your learning needs? (Search)*

• *Were the critical appraisal checklists understandable? (Appraise)*

• *Were the patient case presentations informative? (Integrate)*

##### Attitudes

In the CREATE framework, *Attitude*s refers to the values ascribed by the learner to the importance and usefulness of EBP to inform clinical decision-making. Attitudes are strong predictors of future behaviour [16] and there is emerging evidence that learners' beliefs in the positive benefits of practising EBP are related to the degree with which they implement EBP in their work setting [17]. An example of an attitudes assessment tool is the Evidence-Based Practice Attitude Scale (EBPAS-50); it consists of 50 questions that assess attitude toward EBP and has been validated among mental healthcare and social service providers [18]. For example, the EBPAS-50 [18,19] has survey questions about EBP attitudes answered with a Likert scale ranging from '0' *Not at all* to '4' *To a very great extent*. Respondents rate statements such as:

• *I like to use new types of therapy/interventions to help my clients*.

• *I know better than academic researchers how to care for my clients*.

• *I am willing to use new and different types of therapy/interventions developed by researchers*.

• *Research based treatments/interventions are not clinically relevant*

When assessing attitudes about EBP, it is important to remember that attitudes are hypothesized to be modified by the assessment process [20]. Any tool designed to assess EBP attitudes must consider the manner in which the question is framed. The easiest method of assessing attitudes about EBP may be a written questionnaire. However, it is noted that questionnaires may cause the individual to over-analyse why they hold such attitudes toward the object, thereby distorting their actual attitudes [21]. A more rigorous approach would be to adopt a qualitative methodology during tool development to identify themes on which to base questions, or to triangulate survey data with actual use of an activity in practice [22,23].

##### Self-Efficacy

Within the CREATE framework, *Self-Efficacy *refers to people's judgments regarding their ability to perform a certain activity [24]. For example, an individual's confidence in their ability to search for evidence may be associated with their likelihood to engage in searching [25]. The Evidence-Based Beliefs Scale (EBBS) [26] consists of 16 items that assess confidence in individuals' ability to use EBP (e.g. "I am sure that I can implement EBP") and their beliefs about EBP (e.g. "I believe that EBP results in the best clinical care for patients"). The EBBS demonstrated strong psychometric properties among a large cohort of nurses [17]. Likewise, face and content validity have been reported for the Evidence-based Practice Confidence (EPIC) scale among a variety of healthcare professionals [27].

##### Knowledge

Within the CREATE framework, *Knowledge *refers to learners' retention of facts and concepts about EBP. Hence, assessments of EBP knowledge might assess a learner's ability to define EBP concepts, list the basic principles of EBP, or describe levels of evidence. Knowledge assessment questions might ask learners to identify the most appropriate study design to answer a clinical question or to define Number Needed to Treat. Paper and pencil tests lend themselves well to this level of cognitive assessment. Examples of current EBP knowledge assessment tools are described below.

##### Skills

Within the CREATE framework, *Skills *refer to the application of knowledge, ideally in a practical setting [7]. Assessment of skill would require that learners 'do' a task associated with EBP, such as conduct a search, use a critical appraisal tool to summarize study quality, or calculate Number Needed to Treat. Tools can assess different dimensions of skills, such as the correct application, thoroughness of the process, or the efficiency with which a learner can complete some or all of the processes.

To our knowledge there are two validated instruments that assess a combination of EBP knowledge and skills - the Berlin Questionnaire[28] and the Fresno Test[29]. Both tests ask learners to recall knowledge and describe how they would apply EBP skills in the context of clinical scenarios.

##### Behaviour as Part of Patient Care

Within the CREATE framework, *Behaviour *refers to what learners actually do in practice. It is inclusive of all the processes that a clinician would use in the application of EBP, such as assessing patient circumstances, values, preferences, and goals along with identifying the clinician's own competence relative to the patient's needs in order to determine the focus of an answerable question. EBP-congruent behaviour is essential to translation of EBP-congruent attitudes, knowledge, and skills into benefits for patients and, for assessment, is measured by some form of activity monitoring. The monitored behaviours need to reflect the learning aims and learner audience. Thus, it may be more appropriate for busy front-line clinicians to be monitored on their use of evidence summaries rather than their frequency of searching and appraising primary research [30].

Assessment of EBP behaviour can help to 'lift the lid' on what learners take for granted, and expose the differences between their espoused theories (how they would consciously describe what they do) and their theories in use (what they actually do) [31]. When used for formative purposes, behaviour assessments may help learners identify their learning needs, and help teachers evaluate how well their curriculum equips learners to use EBP in patient care.

Assessing behaviour is not straightforward. Importantly, there may be a Hawthorne effect - the very act of measuring may affect behaviours. Some researchers have assessed behaviour by electronically capturing the searching behaviour of learners [32]. Such approaches, although potentially useful in themselves, cannot fully assess the EBP-congruence of a learner's behaviour. For example, they cannot identify clinical questions that were *not *pursued, or even recognised, and they may not capture what was done with information that was found.

Melnyk and Fineout-Overholt developed and validated the EBP Implementation Scale which assesses learners' self-report of attempts to implement EBP in the workplace [26]. Although Shaneyfelt et al. [4] note the potential biases in retrospective self-reporting behaviours, rigorous critical reflection may address this. Learning portfolios provide a potential strategy for reflection about and evaluation of EBP implementation [33,34], but can be time consuming to complete and assess, and require additional skills in reflective practice. Portfolio use in summative assessment, especially 'high-stakes' assessments, is currently open to question and research is needed to develop these and/or alternative tools [4]. The broader science and theory of behavior change may provide useful alternatives for measuring changes in EBP behaviors [35].

The lines between assessment of knowledge, skills, and behavior can be difficult to discern. Table 2 delineates these three elements of the CREATE framework to facilitate consistent classification of current and future assessment tools.

**Table 2:** Examples of EBP Knowledge, Skills, and Behaviour

Example EBP Construct	Knowledge = Fact Retention	Skills = Performance	Behaviour = Action in Practice
**Searching**	Lists potential databases to search	Modifies search strategy based on preliminary findings	Conducts searches in response to a patient in clinical practice

**Applying evidence summaries**	Aware of high quality evidence summaries	Interprets evidence in context of a unique patient case	Modifies patient care after reviewing an evidence summary

##### Benefit to Patients

Within the CREATE framework, *Benefit to Patients *refers to the impact of EBP educational interventions on the health of patients and communities. The ultimate goal of EBP is to improve care outcomes for patients within the context of complex healthcare systems. Hence, there is a need to assess the impact of EBP education (generally for healthcare providers) on the benefit to patients [9,36]. Measuring benefit to patients as a result of EBP learning is a complex process due to the influence of other variables in the process. In many cases assessment would occur at the institutional level. For example, if all of the care providers on a stroke rehabilitation unit learned how to integrate a clinical practice guideline into their care, would patients on that unit experience better outcomes? This question is intertwined with the much broader issue of how healthcare is delivered. Nevertheless, we propose that it is an important concept to consider within the narrower construct of the outcomes of EBP learning. When a healthcare professional learns to use EBP, we expect that he or she will identify more efficacious care behaviours and ultimately achieve better patient outcomes.

To measure the benefit of EBP for patients, tool developers must identify endpoints in patient care that can be improved through application of EBP. Appropriate endpoints may be different depending on the perspective taken. An individual patient's perspective may be different from the healthcare provider's perspective, and that may be different from a group or an institution's focus on appropriate care endpoints [37]. Measures of individual patient outcomes may include change in a patient's disease state, impairments, functional or social status; their satisfaction with service delivery; or the cost incurred to receive services. Benefit to patients from the healthcare providers' perspective may include change in diagnostic status, functional status, or quality of life. Institutional outcomes may focus on comparisons of service costs with and without EBP, [38] and patient outcomes within diagnostic groupings following implementation of EBP recommendations [39]. Potential endpoints are not specific to steps in the EBP process but rather to patient outcomes, so the CREATE framework does not delineate between the 5-steps for this level of assessment.

Direct measures of patient health outcomes can be derived from clinical documentation to evaluate the impact of EBP approaches. For example, use of EBP approaches are associated with improved outcomes for patients with neck pain [40]; length of stay, overall costs of care, and readmission rates for children with asthma [41]; and the likelihood to prescribe evidence-based interventions in a general hospital [42]. Direct surveys could be used to assess the impact of EBP-based services on patient perceptions about their functional outcomes, health status or satisfaction with services [43]. Care must be taken to avoid wrongful identification of 'best outcomes' based on settings that are easier to study (such as controlled research settings), rather than outcomes with greater ecological validity (such as whole communities) [44]. Additionally, patient care outcomes may need to be measured in conjunction with measures of clinician EBP behaviours to ensure that outcomes can be linked to EBP processes as opposed to other variables that impact patient outcomes.

#### Instruments that Combine Categories

Several tools address more than one category of EBP assessment in a single instrument. The Evidence Based Practice Questionnaire (EBPQ) assesses attitudes, knowledge, and implementation, and has been validated with nurses[45] and social workers [46]. Additionally, the Knowledge, Attitudes, Access and Confidence Evaluation (KACE) has demonstrated adequate discriminative validity, responsiveness to training, and test-retest reliability with dental students and faculty [47].

#### Putting the CREATE Framework to Use

**Figure 3:**
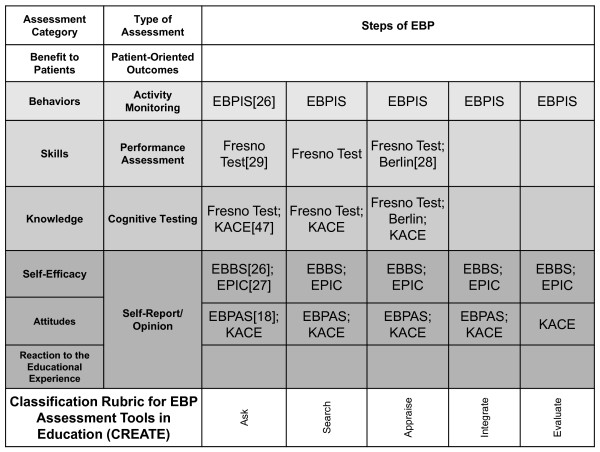
**Classification of validated EBP learning assessment tools using the CREATE framework**. EBPIS: Evidence-Based Practice Implementation Scale; KACE: Knowledge, Attitudes, Access and Confidence Evaluation; EBBS: Evidence-Based Beliefs Scale; EPIC: Evidence-based Practice Confidence Scale; EBPAS: Evidence-Based Practice Attitude Scale.

The need to assess the effectiveness of education and validate the usefulness of EBP approaches is clearly being grappled with by many health professions. As more tools are created, it becomes more important that there is a mechanism for classifying their singular or multiple purposes. The elements of EBP learning assessed by outcome measures referenced in this manuscript are illustrated in the CREATE framework (Figure 3) to demonstrate how the classification process might help developers identify gaps and to help teachers select the best available tools. Tests that are placed within the CREATE model will necessarily need to be weighed in the context of how they are to be used, including who the learners are, the intent of evaluation process, and the environmental contexts in which learning and assessment take place. The CREATE framework is not a model of EBP, but rather it is a tool to classify the intent of EBP educational assessments.

### Part III: Recommendations for EBP assessment tool development

There are substantial needs for development of EBP assessment tools across the categories outlined in this paper: reaction to the educational experience, attitudes, self-efficacy, knowledge, skills, behaviour, and benefit to patients. As noted earlier, assessment tools need to be valid and practical for use by educators and researchers. Validation across learner characteristics (e.g. students vs. clinicians, nurses vs. physicians, users vs. doers) is most useful for broad adoption within EBP education, but as a minimum, tools should identify the type(s) of learner(s) for which they are validated. Guidance for appropriate study design to establish outcome measure validity is beyond the scope of this statement, however many quality references are available [48-50].

Based upon author recommendations and feedback from Sicily 2009 delegates, we propose 4 general recommendations for developers of new EBP learning assessment tools:

1. Use the CREATE framework to classify new tools with regard to EBP steps assessed, assessment category (or categories) addressed, and the audience characteristics and assessment aim for which the tool is intended and/or validated.

2. Clearly state the foundational principles of learning and assessment upon which a new assessment tool is developed.

3. Clearly state how the design of a new tool is linked to the learning aims it is intended to measure.

4. Develop, validate, and use a standardized method for translation of tools into new languages.

Beyond these overarching recommendations, there is need for development of EBP learning assessment tools in each assessment category in the CREATE model:

Reaction to the Educational Experience:

a) A common framework and standardized questions are needed to assess learners' reactions to EBP educational interventions. A standardized assessment would allow reaction to be compared across interventions.

Attitudes and Self-Efficacy:

a) There is a need to build upon existing tools (e.g., EBPAS [19], EBBS[26], EPIC[27], EBPQ[45], KACE[47]) to facilitate measurement of self-reported attitudes, beliefs, and self-efficacy across different learner populations and educational settings.

b) There is a need for reliable qualitative methods to assess EBP attitudes and self-efficacy that can be compared across studies.

Knowledge and Skills:

a) Developers are encouraged to continue psychometric testing of the Fresno Test[29] and Berlin Test[28]) to establish sensitivity to change over time and minimum 'competency' performance for different learner populations and educational settings.

b) The Berlin and Fresno assessments emphasize searching and critical appraisal skill for primary research evidence. Assessments of learners that require different skills are needed (e.g. practitioners that primarily rely on evidence summaries need to be assessed regarding their knowledge of how to appraise and skill for applying evidence summaries and clinical guidelines). 

c) Further investigation is warranted to ascertain ability to obtain and integrate patient values and perspectives in the context of EBP.

d) Assessments that address the performance of EBP skills across clinical environments are needed, including assessment through observation.

Behaviour:

a) Generic self-monitoring tools are needed that measure clinician use of EBP processes in clinical decision-making including, but not limited to: frequency of performing each EBP step, resources used, patient involvement in evidence-based decision-making, frequency of change in clinical management due to newly found evidence, and rate of positive vs. negative outcomes associated with EBP use.

b) Valid, practicable methods are needed for monitoring learners' EBP behaviours that can be used for both formative and summative purposes, particularly 'high stakes' assessments.

Benefit to patients:

a) Tools are needed that measure patient outcomes concurrently with the application of evidence-based approaches to care that inform the impact of EBP behaviours on patient outcomes.

b) Implementation of appropriate qualitative methodologies are needed to determine important outcomes from patients' perspectives with regard to EBP that can be used in diverse healthcare settings.

Finally, within the context of using EBP learning assessment tools in research studies, benefit may be gained from:

1. Using a common set of outcome tools and adopting the operational terms presented in this paper to allow comparison across studies.

2. Including a measure of learners' reaction to the intervention as this may impact effectiveness in other outcome categories.

3. Developing methodologies for assessing the efficacy of interventions designed to target different elements of EBP as defined by the CREATE framework.

4. Assessing the correlation between the assessment categories outlined in the CREATE framework. That is, do the lower order objectives such as attitudes and self-efficacy relate to knowledge and skill and do knowledge and skill relate to behaviour and so on.

## Summary

Evidence-based practice education has spread across professions and clinical settings; however the ability to measure the impact of EBP educational experiences or programs is limited to a few validated tests that do not measure across all levels of learning or steps of EBP. The CREATE framework was developed to provide a classification system for new and existing tools, to help tool developers focus the intent of their tools, and to provide unifying operational definitions to facilitate a common language in EBP learning assessment. We anticipate that use of CREATE to classify EBP learning assessment tools will provide teachers and researchers with an effective method for idenitfying the best available tools for their needs.

We have outlined priorities for EBP assessment tool development generated through an international consensus process and based on the CREATE framework. We hope that consideration of these recommendations will facilitate needed innovations in EBP assessment tool development.

## Competing interests

The authors declare that they have no competing interests.

## Authors' contributions

JKT led development of the manuscript including collection and analysis of delegate feedback. SLK developed the CREATE framework. All authors were involved in drafting the manuscript and in critically revising both the text and the CREATE framework for important intellectual content. All authors read and approved the final manuscript.

## Pre-publication history

The pre-publication history for this paper can be accessed here:

http://www.biomedcentral.com/1472-6920/11/78/prepub
